# Genotype-environment interactions for quantitative traits in Korea Associated Resource (KARE) cohorts

**DOI:** 10.1186/1471-2156-15-18

**Published:** 2014-02-04

**Authors:** Jaemin Kim, Taeheon Lee, Hyun-Jeong Lee, Heebal Kim

**Affiliations:** 1 Interdisciplinary Program in Bioinformatics, Seoul National University, Seoul 151-742, Republic of Korea; 2 Department of Agricultural Biotechnology and Research Institute for Agriculture and Life Sciences, Seoul National University, Seoul 151-742, Republic of Korea; 3 Division of Animal Genomics and Bioinformatics, National Institute of Animal science, #564 Omockchun-dong, Suwon 441-706, Republic of Korea; 4 CHO&KIM Genomics, Seoul National University Research Park, Seoul 151-919, Republic of Korea

**Keywords:** Genotype-environment interaction, Heritability, Obesity, Supra-iliac skinfold thickness

## Abstract

**Background:**

Due to the lack of statistical power and confounding effects of population structure in human population data, genotype-environment interaction studies have not yielded promising results and have provided only limited knowledge for exploring how genotype and environmental factors interact to in their influence onto risk.

**Results:**

We analyzed 49 human quantitative traits in 7,170 unrelated Korean individuals on 326,262 autosomal single nucleotide polymorphisms (SNPs) collected from the KARE (Korean Association Resource) project, and we estimated the statistically significant proportion of variance that could be explained by genotype-area interactions in the supra-iliac skinfold thickness trait ($h_{\text{GE}}^{2}$ = 0.269 and *P* = 0.00032), which is related to abdominal obesity. Data suggested that the genotypes could have different effects on the phenotype (supra-iliac skinfold thickness) in different environmental settings (rural vs. urban areas). We then defined the genotype groups of individuals with similar genetic profiles based on the additive genetic relationships among individuals using SNPs. We observed the norms of reaction, and the differential phenotypic response of a genotype to a change in environmental exposure. Interestingly, we also found that the gene clusters responsible for cell-cell and cell-extracellular matrix interactions were enriched significantly for genotype-area interaction.

**Conclusions:**

This significant heritability estimate of genotype-environment interactions will lead to conceptual advances in our understanding of the mechanisms underlying genotype-environment interactions, and could be ultimately applied to personalized preventative treatments based on environmental exposures.

## Background

Rapid advances in population genetics in recent years have led to significantly improved insight into human complex traits. Although a large number of genetic loci for many complex traits and diseases have been identified using genome-wide association studies (GWAS), the associated variants explain only a small percentage of the overall heritability [1]. Many common, complex traits are a result of the combined effects of genes, environmental factors, and their interactions [2]. Genotype-environment interactions (G×E) were suggested as a possible explanation for “missing heritability” [3], but current knowledge remains insubstantial.

G×E is defined as a phenomenon that phenotypes respond to genotypes differently according to different environmental factors. For example, a specific aspect of the environment might have a greater effect on some genotypes over others. Alternatively, there may be a change in the order of merit of a series of genotypes when they are measured under different environmental conditions [4]. This can be expressed as the norm of reaction (NoR), which represents the profile of phenotypes produced by a genotype across different environments [5]. Reaction norms can be depicted as several curves in two-dimensional graphs, each of which represents the response of a particular genotype to an environmental treatment, and thus the shapes of the NoRs; whether they are parallel or intersect can be used to infer important information regarding G×E [6].

Since G×E can obscure both genetic and environmental effects, the study of G×E is essential for improving accuracy and precision when assessing both genetic and environmental factors [7]. It can also help illustrate how inherited characteristics render some individuals more susceptible to the negative or positive effects of specific environments. This line of investigation is important for identifying mechanisms whereby specific environmental processes might offset or exacerbate genetic risks, thereby suggesting potential targets for preventive interventions [8]. This might ultimately allow us to provide individualized preventative advice before disease diagnosis, based on the knowledge that an individual carries a certain genotype [9].

Despite this importance, there are only a small number of replicated, biologically plausible, and methodologically sound examples of G×E with demonstrated clinical relevance and sufficiently high statistical power [10]. Moreover, previous studies focused on specific genes of interest, rather than considering genome-wide genotype data, and examined variants that were influenced differentially by environmental exposure. For example, Maier (2002) reported that beryllium-exposed workers who are carriers of the Glu69 allele were more likely to develop chronic beryllium lung disease [11]. In addition, Memisoglu et al. (2003) identified a stronger relationship between dietary fat intake and obesity in carriers of the Pro12Ala allele [12]. One classic example of “genome-wide genotype-environment” interaction is J. Clausen’s analysis of the environmental responses of climatic races on Achillea plants. They observed that altitude affected seven distinct genotypes, but not to the same degree or in the same way, by growing genotypically identical plants (clones) in different altitudes at low, medium, and high elevations using cuttings taken from each plant [13]. This direct experiment can be applied only to a species for which genetic replication of a sample is feasible, such as with seed crops.

In the context of the etiology of obesity, single-gene cases cannot account for latent genetic predispositions that are revealed only upon exposure to an obesogenic environment [14]. Instead, obesity is a complex multifactorial phenotype; inter-individual variation in such phenotypes is thought to result from the action of multiple genes and environmental factors. For this reason, the traditional approach to investigating G×E, a locus-specific method, may not be effective for fully delineating the nature and the extent of the genetic polymorphisms involved in obesity-related traits. Therefore, we analyzed how much G×E contributed to variance on a genome-wide scale to better understand the genetic architecture of human complex traits, particularly those involved in obesity. Therefore, our study first estimated the heritability of the G×E component for each trait. Heritability is usually defined as the proportion of total phenotypic variation that is due to additive genetic factors, and thus it is a general and key population parameter that can help in understanding of the genetic architecture of complex traits [15]. The term ‘interaction’ is defined as a departure from additivity in a linear model on a selected measurement scale [16]. As such, statistical interactions are scale dependent; an interaction on the additive scale in a linear regression model may be removable by applying an appropriate transformation [17]. However, to strictly control potential confounders, such as age, gender, and area, in this study, phenotypes were adjusted and transformed for these factors before assessing the significance of G×E. Based on the heritability analysis, we identified an obesity-related trait, in which a G×E component significantly explained the phenotypic variation, and proceeded to perform further analyses including bivariate analysis, norms of reaction, and gene functional classification to elucidate the true genetic basis of human obesity.

## Methods

### Sequence data

The U.S. National Center for Biotechnology Information (NCBI) site was used as the source of the *H. sapiens* genomic sequence (version GRCh37.p5).

### Samples

Data collected by the Korean Association Resource (KARE) project was used for this study. The participants in the KARE project were recruited from two community-based cohorts, Ansung (rural area) and Ansan (urban city), in Gyeonggi Province of South Korea. The Ansung and Ansan cohorts consisted of 5,018 and 5,020 participants, respectively, 40−69 years old and born between 1931 and 1963. This Institutional Review Board of the Korea National Institute of Health approved this study, and all participants provided written informed consent for participation. Based on Cho et al. (2009), we excluded individuals with low call rates (< 96%), sample contamination, gender inconsistencies, cryptic relatedness, and serious concomitant illness, retaining 8,842 subjects (4,183 males and 4,659 females) [18].

### Quality control

The genomic DNA was genotyped on an Affymetrix Genome-Wide Human SNP array 5.0 containing 500,568 SNPs. Markers (GRCh37) with a high missing gene call rate (> 5%), low minor allele frequency (MAF) (<0.01), and significant deviation from the Hardy-Weinberg equilibrium (*P* < 10E−6) were excluded, leaving a total of 326,262 markers to be examined.

### Phenotypes

All individuals were measured for 49 quantitative traits related to obesity, blood pressure, hyperglycemia, diabetes, liver function, lung function, and kidney function. A summary of trait descriptions is provided in Yang et al. (2013) [19]. We adjusted the phenotypes of each trait for the age effect using the model, *y* = *b*_0_ + *b*_1_ × age + *e*, and then standardized the residuals to *z*-scores in each of the cohorts (Ansung and Ansan) and in each gender group separately.

### Environmental factors

We defined three environmental factors in each statistical model: gender, geographical area, and age. For gender, males were coded as A and females as B. The two cities were designated as 1 for Ansung and 2 for Ansan. Age was classified into three different groups: those born in 1931−45 (A), 1946−55 (B), or 1956−63 (C), representing individuals whom experienced the Korean War (1950−53) in their childhood or when older, in their early childhood, and those born after the war, respectively. Most Koreans suffered severe nutritional deficiency during the war. Hypothesizing that nutrition affects phenotypic characteristics, the age groups were defined to determine if different nutritional statuses at a young age interacted with genotypes of a specific trait.

### Additive genetic relationships and unrelated individuals

As implemented in the GCTA (Genome-wide Complex Trait Analysis) tool [20], the genetic relationship between individuals *j* and *k* can be estimated using the following equation:

$$A_{\text{jk}}=\frac{1}{N}\sum_{i=1}^{N}\frac{\left(x_{\text{ij}}-2p_{i}\right)\;\left(x_{\text{ik}}-2p_{i}\right)}{2p_{i}\left(1\;-p_{i}\right)},$$

Where *x*_*ij*_ refers to the number of copies of the reference allele for the *i*^th^ SNP of the *j*^th^ individual, and *p*_*i*_ is the frequency of the reference allele. We estimated the additive genetic relationships between all pairs of individuals from SNP data, and removed one from each pair of individuals with an estimated relatedness > 0.025. Finally, we retained 7,170 “unrelated” individuals for analysis, consisting of 3,261 male and 3,909 female samples and 2,928 rural (Ansung) and 4,242 urban (Ansan) residents (Additional file 1: Table S1). The reason for exclusion was to avoid the estimate of genetic variance being driven by phenotypic correlations for parent-offspring pairs and siblings, which could have then provided a biased estimate of total genetic variance, for example confounding due to shared environmental effects [20].

### G×E estimation and bivariate analysis

To estimate the variance of G×E effects ($\sigma_{\text{ge}}^{2}$), we can specify the mixed linear model (MLM) as **y = Xβ + g + ge + ϵ** with **V = A**_**g**_$\sigma_{g}^{2}$**+ A**_**ge**_$\sigma_{\mathrm{ge}}^{2}$**+ I**$\sigma_{\epsilon }^{2}$, where **g** is an *n* × 1 vector of the aggregate effects of all the autosomal SNPs for all individuals, **A**_**g**_ is the genetic relationship matrix (GRM) between individuals estimated from SNPs, and **ge** is a vector of genotype-environment interaction effects for all individuals, with **A**_**g**_ **= A**_**ge**_ for pairs of individuals in the same environment, and **A**_**ge**_ = 0 for the pairs of individuals in different environments. The environmental effects were fitted as fixed effects in the model: a vector of fixed effects (**β**) with its incidence matrix (X). Because GCTA estimates the variance of the genotype-environment interaction for one environmental factor, three different models were defined separately and analyzed for each environmental factor: gender, age, and area (i.e., gender was fitted as an environmental factor to calculate genotype-gender interactions). The phenotypes were corrected previously for age and gender, and standardized to z-scores in each area cohort data separately to eliminate the necessity to include the other two fixed effects (in this example, age and area). The phenotypic variance ($\sigma_{p}^{2}$) was partitioned into the variance explained by the genetic ($\sigma_{g}^{2}$), G×E ($\sigma_{\text{ge}}^{2}$), and residual variance. The variance explained by all autosomal SNPs by restricted maximum likelihood analysis of MLM was estimated by var(**g**) = **A**_**g**_$\sigma_{g}^{2}$ and var(**ge**) = **A**_**ge**_$\sigma_{\mathrm{ge}}^{2}$, relying on the GRMs. The proportions of variance explained by all autosomal SNPs (narrow-sense heritability) and by G×E were defined as $\sigma_{g}^{2}/\sigma_{p}^{2}$ and $\sigma_{\mathrm{ge}}^{2}/\sigma_{p}^{2}$, respectively. The log-likelihood ratio test (LRT) statistic was calculated to assess the significance of heritability estimates as twice the difference in log-likelihood between the full (h^2^ ≠ 0) and reduced (h^2^ = 0) models, where h^2^ refers to the heritability estimate. The bivariate REML option from this software was used to estimate the genetic correlation between two traits (i.e., SUP in area 1 comprises one trait, and SUP in area 2 comprises the other trait).

### GWAS and functional classification

We used the PLINK-G×E option to test for differences in the association of a trait between two regression coefficients of two different environments using linear regression analysis [21]. The Database for Annotation, Visualization and Integrated Discovery (DAVID) v. 6.7 was used to perform gene functional classification and gene ontology analyses.

## Results and discussion

### Variance explained by a genotype-environment interaction component in the supra-iliac skinfold thickness (SUP) trait

We estimated the proportions of variance that could be explained by genetic and interaction components for each of the 49 traits (Additional file 1: Figure S1 and Tables S2-S4). We examined different environmental factors, such as age, gender, and area. Using a likelihood ratio test for the null hypothesis of **V**_**GE**_ = 0, several traits showed interaction at the level of *P* < 0.05 in each environment (Table 1). Abbreviations of these traits are provided in Additional file 1: Table S4. However, after Bonferroni multiple testing corrections for 147 multiple tests (49 traits × 3 analyses, threshold *P* = 3.4 × 10^-4^), only SUP showed a significant genotype-area interaction ($h_{\text{GE}}^{2}$ = 0.269 and *P* = 0.000315). SUP was measured just above the iliac crest in the mid-axillary line (measured in mm and natural logarithmic transformed) [22], and the anthropometric measurements taken from supra-iliac skinfolds were used to assess abdominal obesity [23]. The histograms of SUP before and after adjustment for age, gender, and area are shown in Additional file 1: Figure S2 in the Supplemental Data. According to our cohort data, females had a higher mean SUP than males (mean = 5.21, SD = 1.20; and mean = 4.58, SD = 1.10, respectively; *P* < 2.2E-16). SUP was also higher in cohorts from the urban city than from rural areas (mean = 5.05; SD = 1.31; mean = 4.82, SD = 1.04, respectively; *P* = 1.1E-14).

**Table 1:** Analysis of genotype-environment interactions (G×E)

**Interaction**	^***a***^**Trait**	***n***	^***b***^**V**_**G**_**/V**_**P**_	**s.e.**	^***c***^**V**_**GE**_**/V**_**P**_	**s.e.**	^***d***^**LRT**	***P***
Genotype × area interaction	HCT	7169	0.003	0.058	0.165	0.080	4.38	1.8E − 02
	RBC	7169	0.105	0.058	0.155	0.080	3.90	2.4E − 02
	SUP	6570	0.000	0.063	0.269	0.087	11.68	3.2E − 04
	WHR	7160	0.014	0.056	0.134	0.076	3.56	3.0E − 02
Genotype × sex interaction	DSS	6753	0.054	0.061	0.157	0.085	3.53	3.0E − 02
	SBP	7169	0.179	0.057	0.145	0.080	3.37	3.3E − 02
	SBP0	7170	0.112	0.058	0.218	0.081	7.19	3.7E − 03
	SONA	7169	0.000	0.056	0.132	0.080	2.77	4.8E − 02
Genotype × age interaction	AST	7169	0.022	0.048	0.148	0.083	3.25	3.6E − 02
	PLAT	7169	0.134	0.050	0.192	0.084	5.36	1.0E − 02
	WBC	7169	0.105	0.050	0.159	0.083	3.70	2.7E − 02

^*a*^*Trait abbreviations*: *HCT* hematocrit, *RBC* red blood cells, *SUP*, supra-iliac skinfold thickness, *WHR* waist-to-hip ratio, *DS* distal radius, *SBP0 and SBP* systolic blood pressure, *SONA* sodium, *AST* aspartate transaminase, *PLAT* platelets, *WBC* white blood cells. ^*b*^The proportion of phenotypic variance explained by the additive genetic effects of all SNPs, *h*^2^_G_ = V_G_/V_P_. ^*c*^The proportion of phenotypic variance explained by additive-by-environment interaction effects of all SNPs. ^*d*^The likelihood ratio test (LRT) for the null hypothesis of V_GE_ = 0, where the LRT statistic is distributed as half the probability of 0 and half the probability of *χ*_1_^2^.

Even though there were some effects of gender and area on the expression of this trait, this does not necessarily contribute to G×E. Nevertheless, the statistically significant heritability estimate of genotype-area interaction from the SUP trait suggests a change in the direction or magnitude of the effect of a genotype in different areas. As such, the genotypes may have a greater or lesser effect on the risk of abdominal obesity in different environmental settings (rural vs. urban area). Together with a very recent study that explored G×E for diabetes-related traits in a European-American population [24], the present study is the first to examine the statistically significant heritability of a G×E on the genome scale.

The proportion of phenotypic variance due to additive genetic effects (V_G_/V_P_) was also estimated from this G×E model (Additional file 1: Tables S2-S4). We compared the estimates of V_G_/V_P_ with or without the G×E component in the model across all 49 traits (Additional file 1: Figure S3). There was a significant positive correlation between Vg estimates (*r* = 0.70 for E as area) with or without G×E in the model. The discrepancy came mostly from the amount of V_G×E_, since the total variance was decomposed into one additional component for the former model, and this caused some difference in estimating the proportion of variance explained by the genetic component.

### Norms of reaction on genotype groups

**Figure 1:**
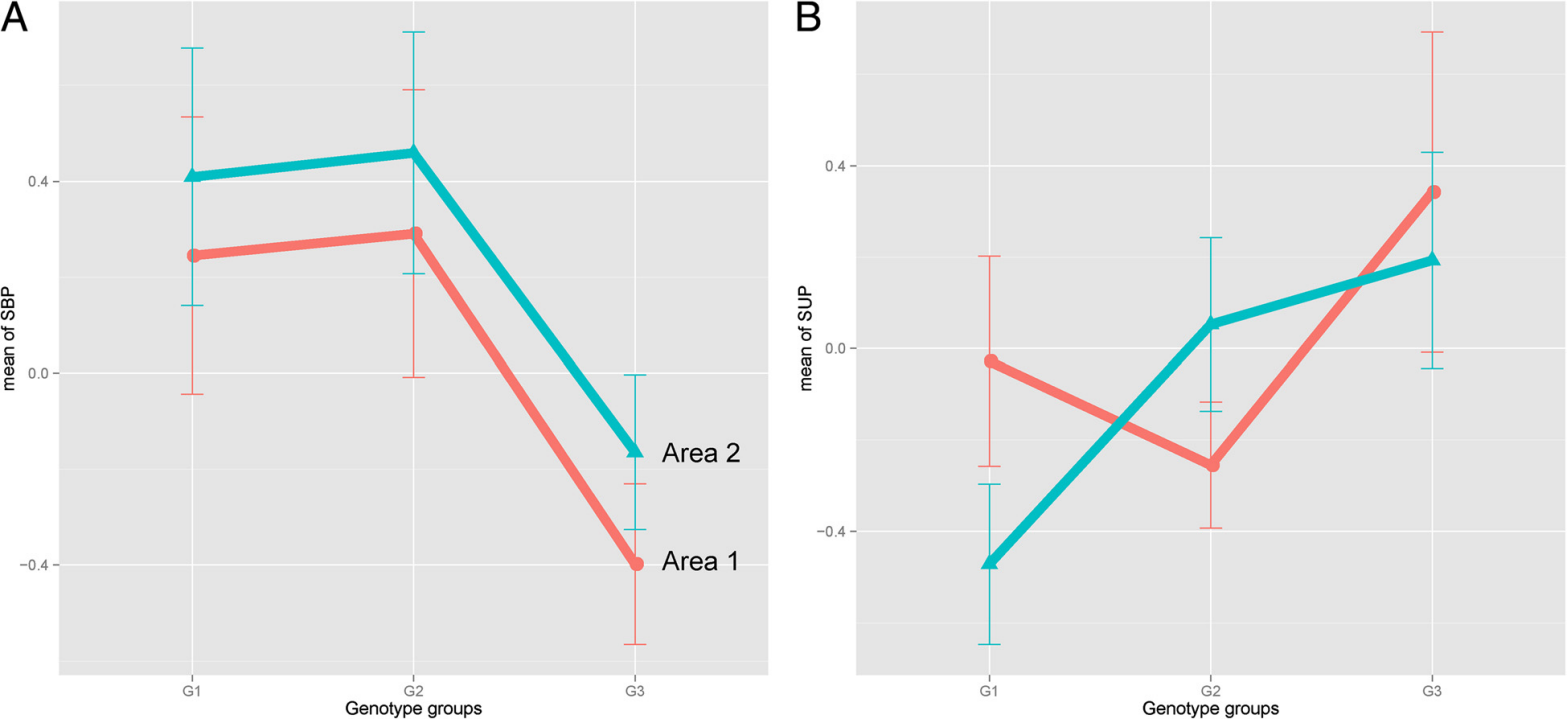
**The norm of reaction.** The responses of two genotype groups to two environmental manipulations (two different cities) were plotted on the same graph such that the phenotypes exhibited by different areas could be compared. Data points represent the mean phenotypic expressions of control (systolic blood pressure; panel **A**) and supra-iliac skinfold thickness **(panel B)** of individuals with similar genotype profiles. Error bars denote the standard errors of the means. Blue lines indicate area 2 (Ansan); red lines represent area 1 (Ansung).

If genotypes can be replicated, and more than one individual of each of several genotypes are reared in different specific environments, then an analysis of variance in a two-way classification of genotype-environment will yield estimates of the variance attributed to the interaction of the genotype with the environment, allowing our results to be quantified and verified [4]. We could then accommodate the concept of NoR. However, this principle cannot be attained in natural human population data, simply because we cannot expect clearly separated genetic distinction in natural populations of sexually-reproducing organisms. Therefore, we instead used the genetic relationship matrix; we cannot replicate the genotypes, but we can find and cluster groups of individuals who share similar genetic components. After exclusion of related pairs (> 0.025) to avoid the possibility of phenotypic resemblance among close relatives caused by non-genetic effects, we defined three different genotype groups from the remaining 7,170 individuals with high genetic relationship values (> 0.020). To achieve this, we first selected the top three individuals who shared close genetic relationships with the largest number of individuals from a given GRM cutoff to accommodate the larger sample size. For example, the first selected individual showed a genetically close relationship with 41 other individuals. Therefore, the first genotype group contained 42 individuals (there were 37 and 36 individuals in the second and third groups, respectively). We finally retained 32, 26, and 25 individuals after excluding samples that belonged to more than one group. The samples used for each genotype group are described in Additional file 1: Table S1. Assuming that individuals from the same genotype group share similar genetic profiles and thus can be hypothetically treated as the same genotype, we attempted to observe how the two different geographical areas impacted genotypes of SUP differently, and we compared this information with the results of a control trait of systolic blood pressure (SBP), in which the interaction component was merely present ($h_{\text{GE}}^{2}$ = 0.000001 and *P* = 0.5) (Figure 1). Phenotypic expression for SBP and SUP was standardized as described above, representing the mean values of all individuals in each genotype group. There were data missing from 600 and one samples for the SUP and SBP traits, respectively, from the KARE project.

Phenotypic values depend on the genotype groups (G) and environmental factors (E) of two areas. For the SBP trait (Figure 1A), G had the main effect, particularly for genotypes 2 and 3; E also had a main effect, but there was no interaction between G and E. In contrast, for the SUP trait (Figure 1B), G and E were found to have main effects and an interaction. The genotypes affected phenotypic values in completely different directions and with different slopes, based on the change in area. This graphical representation supports the fact that SUP has a strong effect on genotype-area interaction compared with the control trait of SBP. However, it must be emphasized that this method is an oversimplification to facilitate and clarify discussion.

### Significant and non-significant genetic variants

We also performed a genome-wide association (GWA) analysis to test genome-wide SNPs for a difference in association between the two environments with SUP and SBP traits [21]. This single SNP association analysis revealed that the most significant SNPs were rs206942 on chromosome 6 (*P* = 2.74E-6) and rs189317 on chromosome 8 (*P* = 8.01E-06) for SUP and SBP, respectively. We confirmed that the associated genetic effect does not necessarily interact with the environment or parity. For example, Cornes et al. found that the fat mass- and obesity-associated common variant (rs9939609 of the *FTO* gene) showed no evidence for G×E [25]. This same variant showed a similar result in our association study: *P*_G_ = 0.001719 and *P*_G×E_ = 0.7766 for the SUP trait, where *P*_G_ and *P*_G×E_ represent the significance of the genotype and genotype-area interaction, respectively. For the SBP trait, rs17249754, known for its relationship with blood pressure [26], had the highest significance for genotype (*P*_G_ = 5.04E-09), but no evidence of genotype-area interaction (*P*_G×E_ = 0.4735).

**Figure 2:**
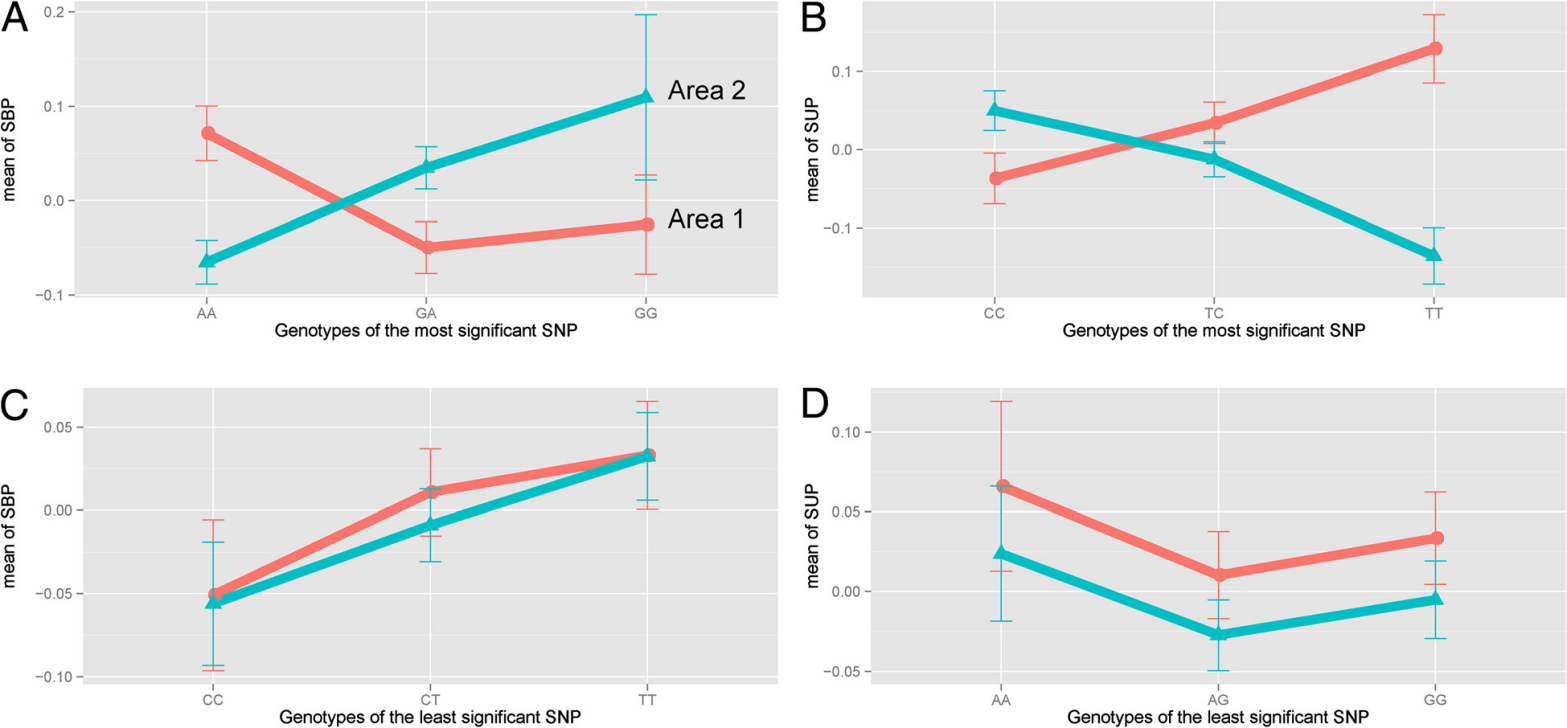
**Locus-specific analysis of the norm of reaction.** The norm of reaction was observed for the most significant loci of the genotype-environment interaction from association studies for both traits **(A and B)**, whereas the parallel shape was seen for the least significant loci of both traits **(C and D)**.

We also identified the least significant SNPs to be rs6063997 on chromosome 20 (*P* = 1) and rs17740112 on chromosome 18 (*P* = 1) for SUP and SBP, respectively. Based on these SNPs, we performed a traditional locus-specific NoR analysis (Figure 2). As expected, for SUP with high G×E effects according to previous results, there was a strong change in direction (Figure 2B) on the most significant locus and a parallel relationship on the least significant SNP (Figure 2D). However, we observed a similar pattern in a control trait of SBP (Figure 2A and C). In addition, the SUP trait had a much larger number of SNPs with *P* < 0.001 that contributed more towards the interaction component than did those for SBP (426 vs. 257 SNPs). These results support the polygenicity of complex traits, in which a few “major” genes together explain only a small fraction of the heritability. There may be a significant locus related to the G×E of a certain trait, but this single locus (or a small number of loci) do not characterize the trait itself. This reveals the limitation of locus-specific analysis for understanding the genetic predisposition of complex traits.

### Bivariate analyses

To determine if variances captured by SNPs differ between areas, we performed bivariate analysis, considering SUP (or SBP as control) in area 1 as one trait and SUP (SBP) in area 2 as the other trait (Additional file 1: Table S6). For SBP, the genetic correlation between areas was 1.00 (s.e. = 0.31), suggesting that the same genetic signals explained the variance in SBP in different areas. In contrast, there was a negative genetic correlation (r_g_ = -0.26, s.e. = 0.26) between areas for the SUP trait, suggesting that the genetic factors for this trait in two areas are not positively correlated (*P* = 0.002, rejecting the null hypothesis r_g_ = 1). Although the significance does not survive multiple testing correction, this result may imply that different genetic signals are associated with abdominal obesity in different areas.

### Gene functional classification and gene ontology analysis

**Figure 3:**
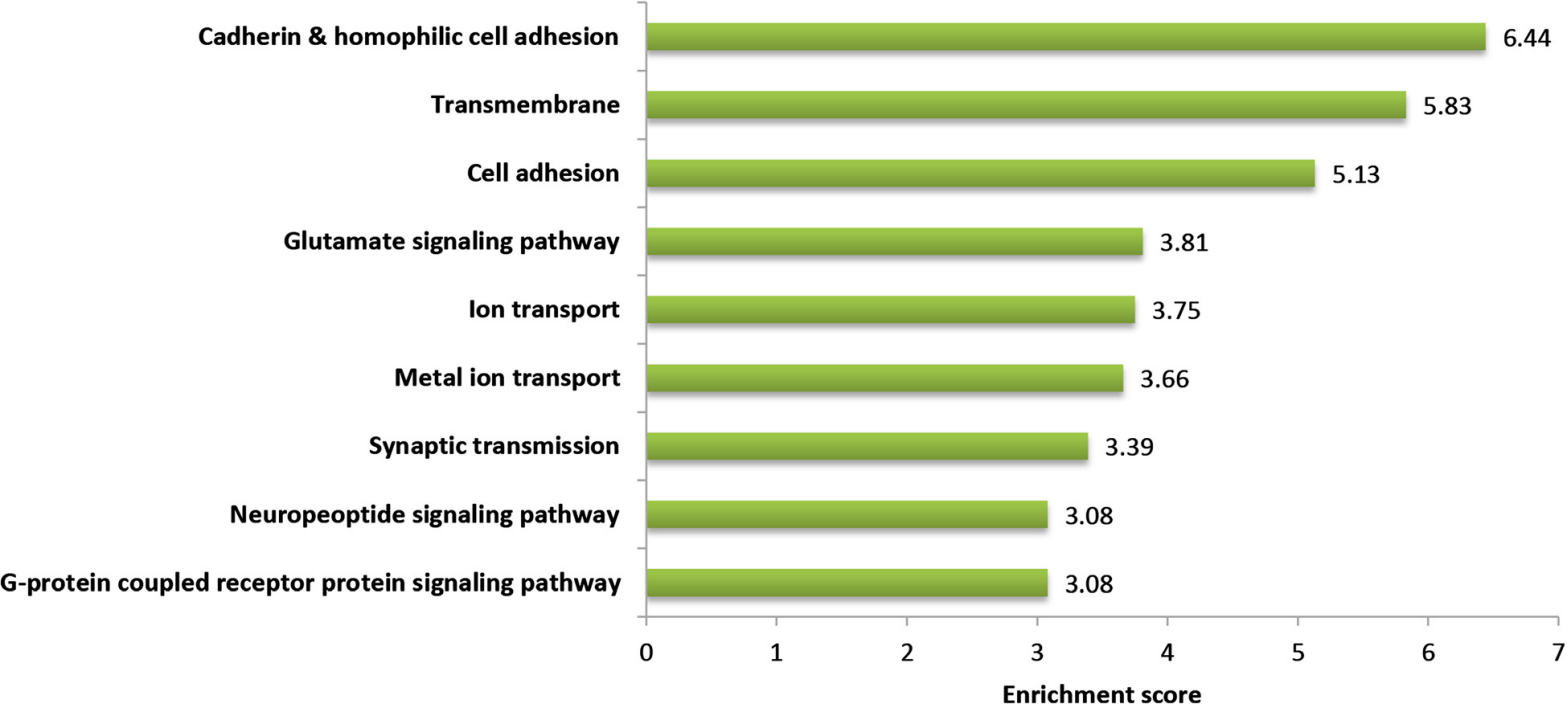
**Gene functional classification of SUP enriched genes.** Nine DAVID gene functional classifications with an enrichment score >3 were selected. The representative terms associated with each cluster were selected manually.

We extracted a total of 1,793 genes from SNPs that exceeded the threshold of *P* < 0.01. We then used the DAVID tool, which clusters functionally related genes together as a unit based on their annotation term co-occurrence, to perform gene functional classification. This allowed us to focus on the larger biological network, rather than on individual genes [27]. The gene functional classification tool clustered genes into nine groups based on the highest stringency and an enrichment score of 3 (which is equivalent to a non-log scale of 0.001). Interestingly, clusters that enriched for genotype-area interactions for SUP were related mostly to functions in cell-cell and cell-extracellular matrix interactions (Figure 3). The ability of cells to communicate with one another and interact with the environment is the hallmark of multicellular organisms. There are several cell communicating mechanisms: cell surface receptors, such as chemically gated ion channels (enrichment scores of 3.75 and 3.66 for ion and metal ion transport, respectively) and G-protein-linked receptors (enrichment score of 3.08), and physical contact with other cells via desmosomes, such as cadherins (enrichment score of 6.44). These interactions are known to influence a number of important cellular activities, including differentiation and proliferation [28]. The cell surface receptors convert an extracellular signal into an intracellular one, responding to the binding of the signal molecule by producing a change within the cellular cytoplasm [29]. Our results showed that, while we still do not understand the underlying biological processes, cells interact with each other and the extracellular matrix differently, based on their exposure to different environmental events; this affected the outcome of SUP as a result of genotype-area interactions. The amount of fat in the body is regulated as part of the energy homeostasis process. In the brain, adiposity signals are integrated with signals from the gastrointestinal system to control energy homeostasis [30]. The brain then responds to the endocrine signals via integrated neuropeptide pathways [31]. This was identified as a cluster (enrichment score of 3.08) related to motor behavior (for example eating or exercise). We also observed that the response to nutrients (GO:0007584), the biological process that results in a change in the state or activity of a cell or organism (via processes such as movement, secretion, enzyme production, or gene expression) as a result of a nutrient stimulus [32], was enriched with 14 genes (*P* = 2.50E-02). The prevalence of obesity was reported to be 25−42% in urban areas, compared with 10−22% in rural areas. The main causes are nutrition (increased consumption of saturated fats and sugars), a sedentary lifestyle, and mechanization. Therefore, rural and urban living might be sufficiently strong environmental exposures that demonstrate large interaction effects with genetic factors [33,34].

### Lack of statistical power in heritability estimates

G×E might not appear in heritability estimates due to the lack of statistical power, particularly if a small fraction of individuals experience adverse exposure, and population stratification in the opposite direction of the allelic effect [35]. However, this specific analysis was exempt from these limitations, since the environmental factor (area) divided the population into approximately equal sample sizes. In addition, we concluded from a previous study [36] and PCA plot Yang et al. [2013]) [19] that the population structure of KARE could be disregarded, and thus did not preclude our analysis of the interaction.

## Conclusions

Although the ‘nature versus nurture’ debate forced the admission that both genetic and environmental factors contribute to phenotypic variation, scientists continue to consider their interaction. Most genetic epidemiology studies have not considered G×E effects, simply because of the difficulty in assessing these effects in quantitative genetic models and the lack of sufficient statistical power to provide sufficient proof [37]. However, based on the findings of the current study, the lifestyle and environmental factors associated with increased risk of obesity could eventually be specified for each individual, and preventive medical and public health strategies could be developed for population subgroups with an emphasis on high-risk individuals. We observed that the SUP trait contains a significant G×E component. This result may be of paramount importance due to increasing evidence that obesity is reaching epidemic proportions worldwide.

## Abbreviations

G×E: Genotype-environment interaction; NoR: Norm of reaction; SUP: Supra-iliac skinfold thickness; SBP: Systolic blood pressure; SNP: Single nucleotide polymorphism; KARE: Korean Association Resource; GCTA: Genome-wide Complex Trait Analysis.

## Competing interests

The authors declare no competing interests.

## Authors’ contributions

JK designed the study, analyzed the data, and wrote the manuscript. TL performed the data analysis. HL and HK conceived and designed the analysis. All authors read, commented on, and approved the manuscript.

## Supplementary Material

Additional file 1: Figure S1 Histograms of the distribution of V_GE_ across 49 traits for each environmental factor area **(A)**, gender **(B)**, and age **(C)**. **Figure S2.** Histograms of the SUP trait before **(A)** and after age, gender, and area adjustment **(B)**. **Figure S3.** The proportion of phenotypic variance due to the additive genetic effects (V_G_/V_P_) with or without G×E in the model. The environmental factor E was defined as area **(A)**, gender **(B)**, or age **(C)**. **Table S1.** Summary of the number of samples used in the analyses. **Table S2.** Variance explained by the genotype-area interaction for 49 traits. **Table S3.** Variance explained by genotype-gender interaction for 49 traits. **Table S4.** Variance explained by genotype-age interaction for all 49 traits. **Table S5.** Abbreviations of the significant traits. **Table S6.** The genetic variances proportional to the total variances (*h*^2^) and the genetic correlation (r_g_) estimated from bivariate analyses using the GCTA tool. Click here for file

## References

[B1] Manolio TA Collins FS Cox NJ Goldstein DB Hindorff LA Hunter DJ McCarthy MI Ramos EM Cardon LR Chakravarti A Finding the missing heritability of complex diseases Nature 2009 461 7265 747 753 10.1038/nature08494 19812666 PMC2831613

[B2] Murcray CE Lewinger JP Gauderman WJ Gene-environment interaction in genome-wide association studies Am J Epidemiol 2009 169 2 219 226 19022827 10.1093/aje/kwn353 PMC2732981

[B3] Frazer KA Murray SS Schork NJ Topol EJ Human genetic variation and its contribution to complex traits Nat Rev Genet 2009 10 4 241 251 19293820 10.1038/nrg2554

[B4] Falconer DS Mackay TFC Frankham R Introduction to quantitative genetics (4th edn) Trends Genet 1996 12 7 280 10.1016/0168-9525(96)81458-2

[B5] Via S Lande R Genotype-environment interaction and the evolution of phenotypic plasticity Evol 1985 39 3 505 522 10.2307/2408649 28561964

[B6] Fuller T Sarkar S Crews D The use of norms of reaction to analyze genotypic and environmental influences on behavior in mice and rats Neurosci Biobehav Rev 2005 29 3 445 456 10.1016/j.neubiorev.2004.12.005 15820549

[B7] Ottman R Gene–environment interaction: definitions and study designs Prev Med 1996 25 6 764 10.1006/pmed.1996.0117 8936580 PMC2823480

[B8] Leve LD Kerr DCR Shaw D Ge X Neiderhiser JM Scaramella LV Reid JB Conger R Reiss D Infant pathways to externalizing behavior: evidence of Genotype × Environment interaction Child Dev 2010 81 1 340 356 10.1111/j.1467-8624.2009.01398.x 20331671 PMC2845990

[B9] Hunter DJ Gene–environment interactions in human diseases Nat Rev Genet 2005 6 4 287 298 15803198 10.1038/nrg1578

[B10] Dempfle A Scherag A Hein R Beckmann L Chang-Claude J Schäfer H Gene–environment interactions for complex traits: definitions, methodological requirements and challenges Eur J Hum Genet 2008 16 10 1164 1172 10.1038/ejhg.2008.106 18523454

[B11] Maier LA Genetic and exposure risks for chronic beryllium disease Clin Chest Med 2002 23 4 827 10.1016/S0272-5231(02)00029-1 12516537

[B12] Memisoglu A Hu FB Hankinson SE Manson JAE De Vivo I Willett WC Hunter DJ Interaction between a peroxisome proliferator-activated receptor γ gene polymorphism and dietary fat intake in relation to body mass Hum Mol Genet 2003 12 22 2923 2929 10.1093/hmg/ddg318 14506127

[B13] Clausen J Keck D Hiesey W Experimental studies on the nature of species. III. Environresponses of climatic races of Achillea Experimental Studies on the Nature of Species III Environresponses of Climatic Races of Achillea 1948 Carnegie Institution of Washington Publ. 581

[B14] Bouchard C Gene–environment interactions in the etiology of obesity: defining the fundamentals Obesity 2008 16 S5 S10 19037213 10.1038/oby.2008.528

[B15] Visscher PM Hill WG Wray NR Heritability in the genomics era—concepts and misconceptions Nat Rev Gen 2008 9 4 255 266 10.1038/nrg2322 18319743

[B16] Satagopan JM Elston RC Evaluation of removable statistical interaction for binary traits Stat Med 2012 32 7 1164 1190 23018341 10.1002/sim.5628 PMC3744333

[B17] An P Mukherjee O Chanda P Yao L Engelman CD Huang CH Zheng T Kovac IP Dubé MP Liang X The challenge of detecting epistasis (G × G interactions): genetic analysis workshop 16 Gen Epidemiol 2009 33 S1 S58 S67 10.1002/gepi.20474 PMC3692280 19924703

[B18] Cho YS Go MJ Kim YJ Heo JY Oh JH Ban H-J Yoon D Lee MH Kim D-J Park M A large-scale genome-wide association study of Asian populations uncovers genetic factors influencing eight quantitative traits Nat Gen 2009 41 5 527 534 10.1038/ng.357 19396169

[B19] Yang J Lee T Kim J Cho M-C Han B-G Lee J-Y Lee H-J Cho S Kim H Ubiquitous polygenicity of human complex traits: genome-wide analysis of 49 traits in Koreans PLoS Gen 2013 9 3 e1003355 10.1371/journal.pgen.1003355 PMC3591292 23505390

[B20] Yang J Lee SH Goddard ME Visscher PM GCTA: a tool for genome-wide complex trait analysis Am J Hum Gen 2011 88 1 76 82 10.1016/j.ajhg.2010.11.011 PMC3014363 21167468

[B21] Purcell S Neale B Todd-Brown K Thomas L Ferreira MA Bender D Maller J Sklar P De Bakker PI Daly MJ PLINK: a tool set for whole-genome association and population-based linkage analyses Am J Hum Gen 2007 81 3 559 575 10.1086/519795 PMC1950838 17701901

[B22] Durnin J Womersley J Body fat assessed from total body density and its estimation from skinfold thickness: measurements on 481 men and women aged from 16 to 72 years Br J Nutr 1974 32 01 77 97 10.1079/BJN19740060 4843734

[B23] Schapira DV Kumar NB Lyman GH Cox CE Abdominal obesity and breast cancer risk Ann Intern Med 1990 112 3 182 186 10.7326/0003-4819-112-3-182 2297194

[B24] Zheng J-S Arnett DK Lee Y-C Shen J Parnell LD Smith CE Richardson K Li D Borecki IB Ordovás JM Genome-wide contribution of genotype by environment interaction to variation of diabetes-related traits PLoS One 2013 8 10 e77442 10.1371/journal.pone.0077442 24204828 PMC3810463

[B25] Cornes B Lind P Medland S Montgomery G Nyholt D Martin N Replication of the association of common rs9939609 variant of FTO with increased BMI in an Australian adult twin population but no evidence for gene by environment (G×E) interaction Int J Obesity 2008 33 1 75 79 10.1038/ijo.2008.223 19030008

[B26] Levy D Ehret GB Rice K Verwoert GC Launer LJ Dehghan A Glazer NL Morrison AC Johnson AD Aspelund T Genome-wide association study of blood pressure and hypertension Nat Genet 2009 41 6 677 687 10.1038/ng.384 19430479 PMC2998712

[B27] Huang DW Sherman BT Lempicki RA Systematic and integrative analysis of large gene lists using DAVID bioinformatics resources Nat Protoc 2009 4 1 44 57 19131956 10.1038/nprot.2008.211

[B28] Albelda SM Buck CA Integrins and other cell adhesion molecules FASEB J 1990 4 11 2868 2880 2199285

[B29] Raven P Johnson G Biology 6th ed 2002 NY: McGraw-Hill Publishing

[B30] Schwartz MW Woods SC Porte D Seeley RJ Baskin DG Central nervous system control of food intake Nat London 2000 404 6778 661 671 10766253 10.1038/35007534

[B31] Woods S Seeley R Understanding the physiology of obesity: review of recent developments in obesity research Int J Obes Relat Metab Disord 2002 26 S8 10.1038/sj.ijo.0802211 12457292

[B32] Ashburner M Ball CA Blake JA Botstein D Butler H Cherry JM Davis AP Dolinski K Dwight SS Eppig JT Gene ontology: tool for the unification of biology Nat Genet 2000 25 1 25 10.1038/75556 10802651 PMC3037419

[B33] Ebrahim S Kinra S Bowen L Andersen E Ben-Shlomo Y Lyngdoh T Ramakrishnan L Ahuja R Joshi P Das SM The effect of rural-to-urban migration on obesity and diabetes in India: a cross-sectional study PLoS Med 2010 7 4 e1000268 10.1371/journal.pmed.1000268 20436961 PMC2860494

[B34] Taylor A Sandeep M Janipalli C Giambartolomei C Evans D Kranthi Kumar M Vinay D Smitha P Gupta V Aruna M Associations of FTO and MC4R variants with obesity traits in Indians and the role of rural/urban environment as a possible effect modifier J Obes 2011 2011 7 10.1155/2011/307542 PMC3139181 21785715

[B35] Eichler EE Flint J Gibson G Kong A Leal SM Moore JH Nadeau JH Missing heritability and strategies for finding the underlying causes of complex disease Nat Rev Genet 2010 11 6 446 450 10.1038/nrg2809 20479774 PMC2942068

[B36] Cho YS Go MJ Kim YJ Heo JY Oh JH Ban HJ Yoon D Lee MH Kim DJ Park M A large-scale genome-wide association study of Asian populations uncovers genetic factors influencing eight quantitative traits Nat Genet 2009 41 5 527 534 10.1038/ng.357 19396169

[B37] Perusse L Bouchard C Genotype‒environment interaction in human obesity Nutr Rev 1999 57 5 31 38 10.1111/j.1753-4887.1999.tb01785.x 10391023

