# Supplementary material for: Genotype-environment interactions for quantitative traits in Korea Associated Resource (KARE) cohorts
Source: BMC Genet. 2014 Feb 4;15:18. doi: 10.1186/1471-2156-15-18 (PMC3922112; doi:10.1186/1471-2156-15-18)
Supplement: Additional file 1: Figure S1 — Histograms of the distribution of VGE across 49 traits for each environmental factor area (A), gender (B), and age (C). Figure S2. Histograms of the SUP trait before (A) and after age, gender, and area adjustment (B). Figure S3. The proportion of phenotypic variance due to the additive genetic effects (VG/VP) with or without G×E in the model. The environmental factor E was defined as area (A), gender (B), or age (C). Table S1. Summary of the number of samples used in the analyses. Table S2. Variance explained by the genotype-area interaction for 49 traits. Table S3. Variance explained by genotype-gender interaction for 49 traits. Table S4. Variance explained by genotype-age interaction for all 49 traits. Table S5. Abbreviations of the significant traits. Table S6. The genetic variances proportional to the total variances (h2) and the genetic correlation (rg) estimated from bivariate analyses using the GCTA tool. [file 1471-2156-15-18-S1.docx]

**Genotype-environment interaction for quantitative traits in Korea Associated Resource (KARE) cohorts**

Jaemin Kim, Taeheon Lee, Hyun-Jeong Lee, and Heebal Kim

| **SUPPLEMENTARY INFORMATION** |
| --- |

1. **Supplementary Figures**

**Supplementary Figure 1……………………………………………………..2**

**Supplementary Figure 2……………………………………………………..3**

**Supplementary Figure 3……………………………………………………..4**

1. **Supplementary Tables**

**Supplementary Table 1……………………………………………………….5**

**Supplementary Table 2……………………………………………………….6**

**Supplementary Table 3……………………………………………………….8**

**Supplementary Table 4……………………………………………………….10**

**Supplementary Table 5……………………………………………………….12**

**Supplementary Table 6……………………………………………………….13**

**3. Supplementary References**

**Figure S1.** Histograms of the distribution of V_GE_ across 49 traits for each environmental factor area (A), gender (B), and age (C).

**
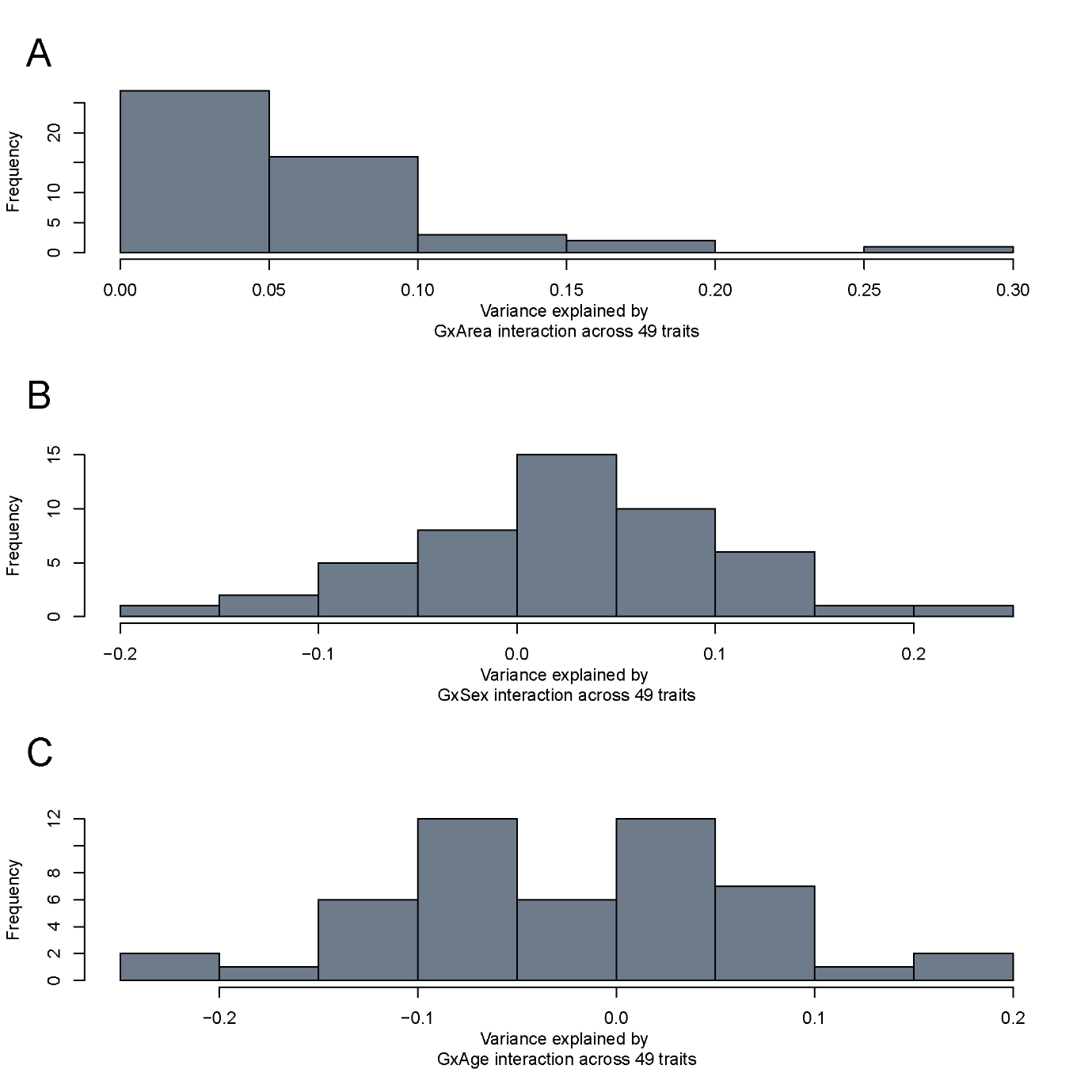
**

**Figure S2.** Histograms of the SUP trait before (A) and after age, gender, and area adjustment (B).

**
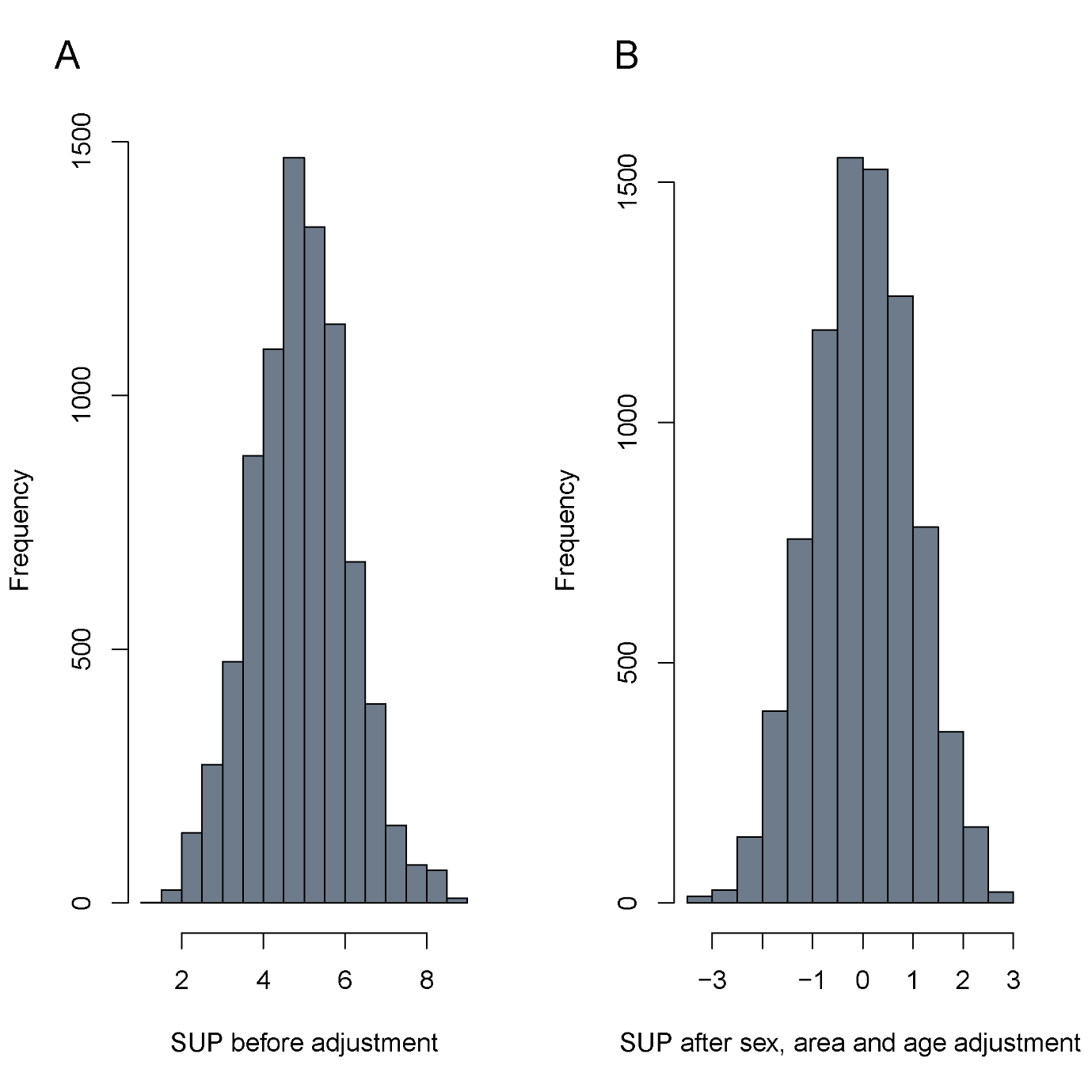
**

**Figure S3.** The proportion of phenotypic variance due to the additive genetic effects (V_G_/V_P_) with or without G×E in the model. The environmental factor E was defined as area (A), gender (B), or age (C).

**
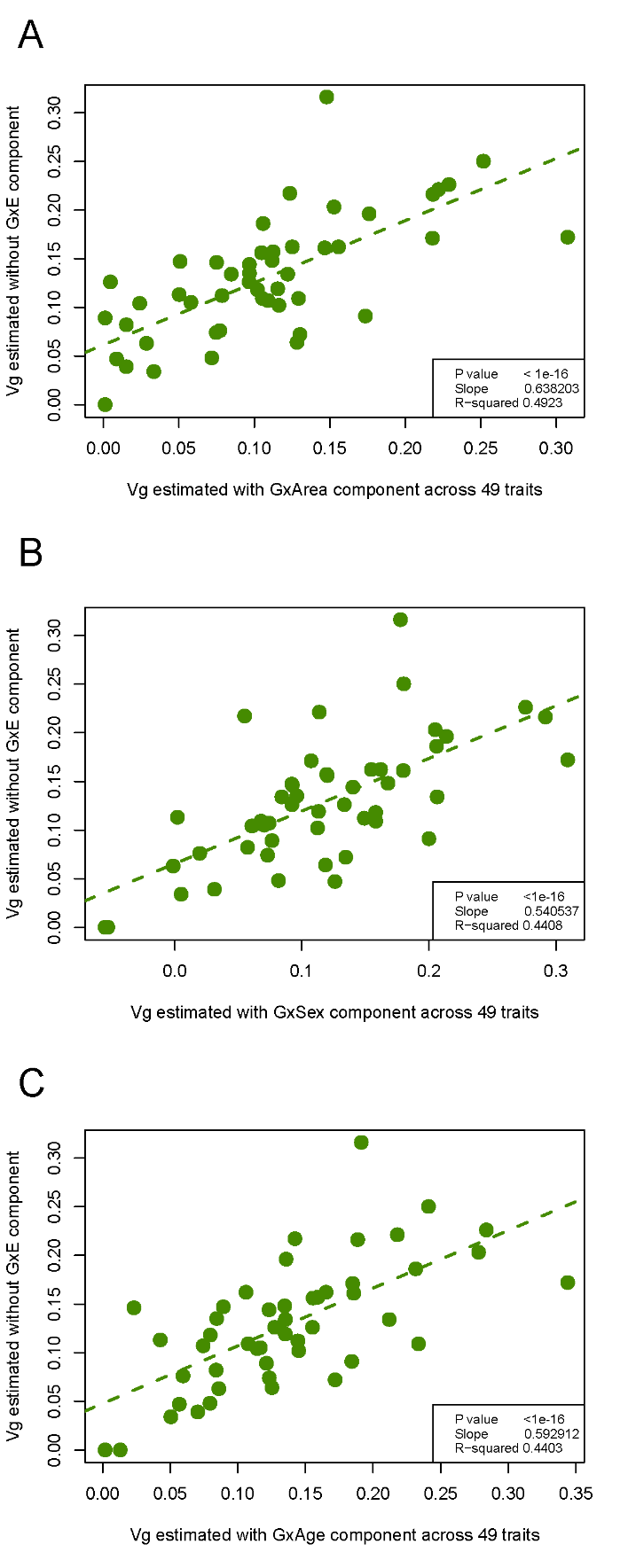
**

**Table S1**. Summary of the number of samples used in the analyses.

|  | Samples  (n) | Genotype  Group 1 | Genotype  Group 2 | Genotype  Group 3 |
| --- | --- | --- | --- | --- |
| Ansung (Area 1) | 2,928 | 22 | 12 | 10 |
| Ansan (Area 2) | 4,242 | 10 | 14 | 15 |
| Total | 7,170 | 32 | 26 | 25 |

**Table S2**. Variance explained by the genotype-area interaction for 49 traits.

| Traits | V_G_/V_P_ | V_GE_/V_P_ | LRT | *P* | n |
| --- | --- | --- | --- | --- | --- |
| ALT | 0.146785 | 0.000001 | 0 | 0.5 | 7169 |
| AST | 0.073837 | 0.000001 | 0 | 0.5 | 7169 |
| BMI | 0.129114 | 0.036083 | 0.214 | 0.322 | 7168 |
| Bun | 0.049672 | 0.103753 | 1.743 | 0.0934 | 7169 |
| CHL | 0.115137 | 0.000001 | 0 | 0.5 | 7169 |
| Creatine | 0.04907 | 0.000001 | 0 | 0.5 | 7169 |
| CRP | 0.070645 | 0.074032 | 0.895 | 0.172 | 7168 |
| DBP | 0.128139 | 0.082125 | 1.208 | 0.136 | 7170 |
| DBP0 | 0.216881 | 0.000001 | 0 | 0.5 | 7169 |
| DT | 0.122354 | 0.024766 | 0.092 | 0.381 | 6753 |
| GLU0 | 0.09562 | 0.0315 | 0.17 | 0.34 | 7006 |
| GLU120 | 0.077509 | 0.079448 | 1.057 | 0.152 | 6830 |
| GLU60 | 0.100945 | 0.005497 | 0.005 | 0.472 | 6824 |
| HB | 0.022901 | 0.077836 | 0.927 | 0.168 | 7169 |
| HBA1C | 0.127097 | 0.000001 | 0 | 0.5 | 7168 |
| HCT | 0.00348 | 0.165485 | 4.38 | 0.0182 | 7169 |
| HDL | 0.172453 | 0.000001 | 0 | 0.5 | 7169 |
| Height | 0.306452 | 0.020233 | 0.067 | 0.398 | 7170 |
| Hip | 0.09542 | 0.061086 | 0.578 | 0.223 | 7160 |
| HOMA | 0.000001 | 0.060687 | 0.08 | 0.389 | 7006 |
| INS0 | 0.000001 | 0.050087 | 0 | 0.5 | 7007 |
| INS120 | 0.095587 | 0.094874 | 1.312 | 0.126 | 6824 |
| INS60 | 0.073592 | 0.000001 | 0 | 0.5 | 6823 |
| LDL | 0.083587 | 0.099716 | 1.606 | 0.102 | 6963 |
| MT | 0.10789 | 0.000001 | 0 | 0.5 | 6771 |
| NONHDL | 0.111297 | 0.090988 | 1.457 | 0.114 | 7169 |
| pH | 0.014031 | 0.048837 | 0.407 | 0.262 | 7147 |
| PLAT | 0.174954 | 0.040522 | 0.273 | 0.301 | 7169 |
| POTA | 0.007553 | 0.079022 | 1.025 | 0.156 | 7169 |
| Pulse | 0.114336 | 0.010446 | 0.018 | 0.446 | 7162 |
| RBC | 0.104654 | 0.155187 | 3.898 | 0.0242 | 7169 |
| RENIN | 0.076048 | 0.000001 | 0 | 0.5 | 7169 |
| RGTP | 0.10423 | 0.008917 | 0.015 | 0.451 | 7169 |
| SBP | 0.250584 | 0.000001 | 0 | 0.5 | 7169 |
| SBP0 | 0.220922 | 0.000001 | 0 | 0.5 | 7170 |
| SG | 0.032265 | 0.003861 | 0.003 | 0.48 | 7147 |
| SONA | 0.027406 | 0.069317 | 0.722 | 0.198 | 7169 |
| SP1 | 0.227816 | 0.000001 | 0 | 0.5 | 7009 |
| SP2 | 0.120982 | 0.024328 | 0.093 | 0.38 | 7007 |
| SP3 | 0.110658 | 0.071785 | 0.788 | 0.187 | 7011 |
| SUB | 0.151643 | 0.098659 | 1.407 | 0.118 | 7138 |
| SUP | 0.000001 | 0.268978 | 11.683 | 0.000315 | 6570 |
| TCHL | 0.103605 | 0.103118 | 1.859 | 0.0864 | 7169 |
| TG | 0.21721 | 0.000001 | 0 | 0.5 | 7169 |
| THDL | 0.15469 | 0.014679 | 0.039 | 0.422 | 7169 |
| Waist | 0.056798 | 0.098304 | 1.748 | 0.0931 | 7163 |
| WBC | 0.123995 | 0.074321 | 0.933 | 0.167 | 7169 |
| Weight | 0.145557 | 0.030669 | 0.154 | 0.347 | 7168 |
| WHR | 0.014067 | 0.134405 | 3.56 | 0.0296 | 7160 |

*^a^* proportion of phenotypic variance explained by the additive genetic effects of all SNPs, *h*^2^_G_ = V_G_ / V_P_. *^b^* proportion of phenotypic variance explained by additive-by-environment interaction effects of all SNPs. *^c^* likelihood ratio test (LRT) for the null hypothesis of V_GE_ = 0, where the LRT statistic is distributed as half probability of 0 and half probability of *χ*_1_^2^.

**Table S3**. Variance explained by genotype-gender interaction for 49 traits.

| Traits | V_G_/V_P_ | V_GE_/V_P_ | LRT | *P* | n |
| --- | --- | --- | --- | --- | --- |
| ALT | 0.176269 | -0.059324 | 0.554 | 0.228 | 7169 |
| AST | 0.09121 | -0.037312 | 0.227 | 0.317 | 7169 |
| BMI | 0.133192 | 0.026048 | 0.118 | 0.365 | 7168 |
| BUN | 0.090756 | 0.024045 | 0.097 | 0.378 | 7169 |
| CHL | 0.11103 | 0.004149 | 0.003 | 0.478 | 7169 |
| Creatine | 0.000818 | 0.090293 | 1.304 | 0.127 | 7169 |
| CRP | 0.080328 | 0.054341 | 0.488 | 0.243 | 7168 |
| DBP | 0.156762 | 0.122491 | 2.408 | 0.0603 | 7169 |
| DBP0 | 0.106005 | 0.129681 | 2.541 | 0.0555 | 7170 |
| DS | 0.053633 | 0.157432 | 3.531 | 0.0301 | 6753 |
| GLU0 | 0.09473 | 0.033678 | 0.175 | 0.338 | 7006 |
| GLU120 | 0.147837 | -0.058332 | 0.481 | 0.244 | 6830 |
| GLU60 | 0.156783 | -0.099942 | 1.434 | 0.116 | 6824 |
| HB | 0.059435 | 0.008418 | 0.011 | 0.458 | 7169 |
| HBA1C | 0.117218 | 0.017164 | 0.049 | 0.412 | 7168 |
| HCT | 0.090843 | -0.000161 | 0 | 0.499 | 7169 |
| HDL | 0.198651 | -0.052312 | 0.488 | 0.242 | 7169 |
| Height | 0.307796 | 0.01654 | 0.045 | 0.416 | 7170 |
| Hip | 0.132071 | -0.011633 | 0.023 | 0.439 | 7160 |
| HOMA | -0.05608 | 0.08776 | 1.162 | 0.14 | 7006 |
| INS0 | -0.054 | 0.02899 | 0.132 | 0.358 | 7007 |
| INS120 | 0.138865 | 0.010126 | 0.015 | 0.451 | 6824 |
| INS60 | 0.071754 | 0.003903 | 0.002 | 0.481 | 6823 |
| LDL | 0.082915 | 0.101925 | 1.622 | 0.101 | 6963 |
| MS | 0.072937 | 0.067862 | 0.696 | 0.202 | 6771 |
| NONHDL | 0.118182 | 0.078471 | 1.027 | 0.155 | 7169 |
| pH | 0.029901 | 0.018122 | 0.055 | 0.407 | 7147 |
| PLAT | 0.212475 | -0.034781 | 0.2 | 0.327 | 7169 |
| POTA | 0.124745 | -0.151855 | 3.95 | 0.0234 | 7169 |
| Pulse | 0.111809 | 0.014424 | 0.034 | 0.427 | 7162 |
| RBC | 0.204739 | -0.038095 | 0.246 | 0.31 | 7169 |
| RENIN | 0.018314 | 0.116233 | 2.359 | 0.0623 | 7169 |
| RGTP | 0.066591 | 0.080642 | 1.042 | 0.154 | 7169 |
| SBP | 0.17886 | 0.144971 | 3.368 | 0.0332 | 7169 |
| SBP0 | 0.112404 | 0.218474 | 7.193 | 0.00366 | 7170 |
| SG | 0.003715 | 0.059371 | 0.565 | 0.226 | 7147 |
| SONA | -0.00256 | 0.133099 | 2.775 | 0.0479 | 7169 |
| SP1 | 0.274696 | -0.096727 | 1.622 | 0.101 | 7009 |
| SP2 | 0.205139 | -0.142687 | 3.529 | 0.0301 | 7007 |
| SP3 | 0.166448 | -0.037678 | 0.232 | 0.315 | 7011 |
| SUB | 0.203588 | -0.001554 | 0 | 0.492 | 7138 |
| SUP | 0.075157 | 0.027163 | 0.101 | 0.376 | 6570 |
| TCHL | 0.118717 | 0.076955 | 0.976 | 0.162 | 7169 |
| TG | 0.29034 | -0.144613 | 4.068 | 0.0218 | 7169 |
| THDL | 0.160732 | 0.002352 | 0.001 | 0.488 | 7169 |
| Waist | 0.069014 | 0.070154 | 0.876 | 0.175 | 7163 |
| WBC | 0.153383 | 0.016187 | 0.045 | 0.416 | 7169 |
| Weight | 0.178453 | -0.036207 | 0.225 | 0.317 | 7168 |
| WHR | 0.055971 | 0.052549 | 0.505 | 0.239 | 7160 |

**Table S4**. Variance explained by genotype-age interaction for all 49 traits.

| Traits | V_G_/V_P_ | V_GE_/V_P_ | LRT | *P* | n |
| --- | --- | --- | --- | --- | --- |
| ALT | 0.190083 | -0.14108 | 3.119 | 0.0387 | 7169 |
| AST | 0.021942 | 0.148018 | 3.25 | 0.0357 | 7169 |
| BMI | 0.17068 | -0.05977 | 0.513 | 0.237 | 7168 |
| BUN | 0.088016 | 0.042983 | 0.264 | 0.304 | 7169 |
| CHL | 0.143725 | -0.08737 | 1.215 | 0.135 | 7169 |
| Creatine | 0.041402 | 0.019126 | 0.056 | 0.407 | 7169 |
| CRP | 0.078193 | 0.089066 | 1.121 | 0.145 | 7168 |
| DBP | 0.232332 | -0.06262 | 0.636 | 0.212 | 7169 |
| DBP0 | 0.183654 | -0.06119 | 0.577 | 0.224 | 7170 |
| DS | 0.140985 | -0.0005 | 0 | 0.498 | 6753 |
| GLU0 | 0.083119 | 0.088428 | 1.067 | 0.151 | 7006 |
| GLU120 | 0.143203 | -0.0738 | 0.751 | 0.193 | 6830 |
| GLU60 | 0.078388 | 0.078823 | 0.851 | 0.178 | 6824 |
| HB | 0.113089 | -0.14199 | 2.751 | 0.0486 | 7169 |
| HBA1C | 0.124009 | 0.004469 | 0.003 | 0.479 | 7168 |
| HCT | 0.126035 | -0.10243 | 1.447 | 0.114 | 7169 |
| HDL | 0.18298 | -0.02892 | 0.124 | 0.362 | 7169 |
| Height | 0.342593 | -0.07961 | 1.068 | 0.151 | 7170 |
| Hip | 0.153919 | -0.07871 | 0.888 | 0.173 | 7160 |
| HOMA | 0.011798 | -0.06822 | 0.674 | 0.206 | 7006 |
| INS0 | 0.000648 | -0.1191 | 2.039 | 0.0766 | 7007 |
| INS120 | 0.121797 | 0.066744 | 0.575 | 0.224 | 6824 |
| INS60 | 0.121976 | -0.14259 | 2.654 | 0.0516 | 6823 |
| LDL | 0.133939 | 0.000994 | 0 | 0.495 | 6963 |
| MS | 0.073164 | 0.092112 | 1.129 | 0.144 | 6771 |
| NONHDL | 0.157943 | 0.002308 | 0.001 | 0.489 | 7169 |
| pH | 0.069124 | -0.08868 | 1.137 | 0.143 | 7147 |
| PLAT | 0.134453 | 0.192037 | 5.36 | 0.0103 | 7169 |
| POTA | 0.0555 | -0.02523 | 0.093 | 0.38 | 7169 |
| Pulse | 0.133883 | -0.04821 | 0.326 | 0.284 | 7162 |
| RBC | 0.230261 | -0.12927 | 2.302 | 0.0646 | 7169 |
| RENIN | 0.058255 | 0.05509 | 0.459 | 0.249 | 7169 |
| RGTP | 0.10642 | 0.011602 | 0.021 | 0.443 | 7169 |
| SBP | 0.239723 | 0.031625 | 0.155 | 0.347 | 7169 |
| SBP0 | 0.216727 | 0.006966 | 0.007 | 0.466 | 7170 |
| SG | 0.049216 | -0.04215 | 0.261 | 0.305 | 7147 |
| SONA | 0.084704 | -0.06406 | 0.574 | 0.224 | 7169 |
| SP1 | 0.282478 | -0.17239 | 4.148 | 0.0208 | 7009 |
| SP2 | 0.210682 | -0.23707 | 8.367 | 0.00191 | 7007 |
| SP3 | 0.133402 | 0.046312 | 0.29 | 0.295 | 7011 |
| SUB | 0.276767 | -0.21939 | 7.132 | 0.00379 | 7138 |
| SUP | 0.119891 | -0.08563 | 0.91 | 0.17 | 6570 |
| TCHL | 0.154344 | 0.009113 | 0.012 | 0.456 | 7169 |
| TG | 0.187412 | 0.095509 | 1.336 | 0.124 | 7169 |
| THDL | 0.163984 | 0.002973 | 0.001 | 0.486 | 7169 |
| Waist | 0.115349 | -0.0172 | 0.042 | 0.419 | 7163 |
| WBC | 0.104764 | 0.158835 | 3.697 | 0.0273 | 7169 |
| Weight | 0.184464 | -0.06374 | 0.56 | 0.227 | 7168 |
| WHR | 0.082737 | 0.013798 | 0.028 | 0.434 | 7160 |

**Table S5.** Abbreviations of the significant traits. A complete list of all 49 traits and their abbreviations are provided in Table S1 of Jian et al. (2013) [[1](#_ENREF_1)].

| Trait | Trait abbreviation |
| --- | --- |
| Supra-iliac skinfold thickness | SUP |
| Systolic blood pressure | SBP0, SBP |
| White blood cells | WBC |
| Red blood cells | RBC |
| Platelets | PLAT |
| Hematocrit | HCT |
| Waist-to-hip ratio | WHR |
| Distal radius | DS |
| Sodium | SONA |
| Aspartate transaminase | AST |

**Table S6**. The genetic variances proportional to the total variances () and the genetic correlation (r_g_) estimated from bivariate analyses using the GCTA tool.

| Trait 1/ Trait 2 | Samples | Trait 1  $h^{2}$ (SE) | Trait 2  $h^{2}$ (SE) | Genetic correlation  r_g_ (SE) | P-value  H_0_: r_g_ = 0 | P-value  H_0_: r_g_ = 1 |
| --- | --- | --- | --- | --- | --- | --- |
| SUP in area 1/ SUP in area 2 | 2927/3642 | 0.31 (0.10) | 0.19 (0.08) | -0.26 (0.26) | 0.2 | 0.002 |
| SBP in area 1/ SBP in area 2 | 2927/4241 | 0.34 (0.10) | 0.18 (0.07) | 1.00 (0.31) | 1e-06 | 0.5 |

**Reference**

1. Yang J, Lee T, Kim J, Cho M-C, Han B-G, Lee J-Y, Lee H-J, Cho S, Kim H: **Ubiquitous Polygenicity of Human Complex Traits: Genome-Wide Analysis of 49 Traits in Koreans**. *PLoS genetics* 2013, **9**(3):e1003355.
